# Diet, Ultra-Processed Foods, and Food Additives in Crohn’s Disease: A Comprehensive Review

**DOI:** 10.3390/nu18162652

**Published:** 2026-08-14

**Authors:** Giovanna Flore, Andrea Deledda, Amalia Di Petrillo

**Affiliations:** Department of Medical Sciences and Public Health, University of Cagliari, 09124 Cagliari, Italy; gflore@unica.it (G.F.); andredele@tiscali.it (A.D.)

**Keywords:** Crohn disease, food additives, ultra-processed food

## Abstract

Background and aim: Crohn’s disease (CD) is a chronic inflammatory disorder of the gastrointestinal tract resulting from a complex interplay among genetic susceptibility, immune dysregulation, environmental factors, and alterations in the gut microbiota. Among these, diet has emerged as a key modifiable factor influencing intestinal inflammation, barrier integrity, and disease progression. This review aims to critically evaluate the role of dietary patterns and food additives in the pathogenesis and management of Crohn’s disease, with particular emphasis on their effects on intestinal inflammation, epithelial barrier function, and host–microbiota interactions. Methods: A comprehensive literature search was conducted using the PubMed, Scopus, and Web of Science databases up to March 2026. Clinical trials, observational studies, and mechanistic research were included based on their relevance to dietary factors, food additives, and intestinal inflammation in CD. Evidence was synthesized narratively to integrate clinical and experimental findings. Results: Dietary interventions such as the Crohn’s disease exclusion diet (CDED), specific carbohydrate diet (SCD), low-FODMAP diet, and Mediterranean diet (MD) demonstrate varying degrees of efficacy in improving clinical symptoms and, in some cases, inflammatory markers. In contrast, high consumption of ultra-processed foods is consistently associated with increased disease risk and activity. Emerging evidence highlights the role of food additives—particularly emulsifiers and artificial sweeteners—in disrupting gut barrier integrity, promoting dysbiosis, and enhancing inflammatory responses. Recent human studies further suggest that exposure to these compounds may correlate with disease activity, supporting their potential role in CD pathophysiology. Conclusions: Diet plays a significant adjunctive role in the management of Crohn’s disease. Reducing exposure to ultra-processed foods and potentially harmful food additives, while promoting balanced dietary patterns, may improve clinical outcomes. Further research is needed to clarify long-term effects and to develop personalized, evidence-based nutritional strategies for patients with CD.

## 1. Introduction

Inflammatory bowel diseases (IBDs) encompass a group of chronic, relapsing–remitting inflammatory conditions of the gastrointestinal (GI) tract, with Crohn’s disease (CD) and ulcerative colitis (UC) being the two primary forms. These diseases are characterized by an aberrant immune response to the intestinal microbiota in genetically predisposed individuals, leading to inflammation, mucosal damage, and GI and extraintestinal symptoms [1].

CD is a type of IBD that can affect any part of the GI tract from the mouth to the anus, though it most commonly impacts the terminal ileum and the colon. CD is particularly challenging to manage due to its unpredictable course and tendency to cause complications such as strictures, fistulas, and abscesses [2]. The etiology of CD is multifactorial, involving genetic predisposition, environmental factors, immune system dysregulation, and, critically, alterations in the gut microbiota [3].

Among these factors, diet has emerged as a crucial modifiable element that can influence both the risk and progression of CD. Increasing evidence suggests that dietary components not only shape gut microbiota composition but also modulate immune responses and affect intestinal permeability, thereby playing a significant role in disease pathogenesis [4,5,6,7].

In addition to overall dietary patterns, increasing attention has been directed toward specific components of modern diets, particularly ultra-processed foods and food additives. These compounds may contribute to intestinal inflammation through multiple mechanisms, including the impairment of epithelial barrier function, the modulation of immune responses, and interactions with the gut microbiome [6].

Dietary patterns strongly influence intestinal homeostasis. Diets rich in fiber and polyphenols, such as the Mediterranean diet (MD), promote the growth of beneficial bacteria and increase the production of short-chain fatty acids (SCFAs), which exert anti-inflammatory effects [8,9]. In contrast, the consumption of ultra-processed foods, high-fat Western diets, and foods rich in animal fats and sugary beverages has been associated with increased intestinal permeability, microbial dysbiosis, and heightened gut inflammation [10]. These diets are also linked to a reduction in SCFA-producing bacteria and an increased abundance of Gram-negative bacteria, contributing to pro-inflammatory signaling [9,11].

Beyond microbiota modulation, diet plays a fundamental role in regulating immune responses. The interaction among dietary components, gut microbes, and immune cells is essential for maintaining intestinal homeostasis. Certain nutrients, including omega-3 fatty acids and polyphenols, promote regulatory T-cell (Treg) activity, enhance barrier integrity, and reduce pro-inflammatory cytokine production. They also support the development of tolerogenic dendritic cells, which secrete anti-inflammatory mediators such as transforming growth factor-beta (TGF-β) and interleukin-10 (IL-10) [12,13].

Conversely, Western dietary patterns have been associated with increased activation of pro-inflammatory immune pathways, leading to elevated levels of cytokines such as tumor necrosis factor-alpha (TNF-α) and interleukin-6 (IL-6). High-fat diets may also impair epithelial mitochondrial function, further contributing to dysbiosis and mucosal inflammation [14,15].

Importantly, while pharmacological therapies remain the cornerstone of treatment, nutritional strategies are increasingly recognized as complementary approaches in the management of CD, with growing evidence supporting their role in modulating disease activity and improving patient outcomes. However, current data remain heterogeneous, and clear clinical guidelines are still lacking. Therefore, the aim of this review is to critically evaluate the current evidence on dietary patterns and food additives in CD, with a particular focus on their effects on intestinal inflammation, barrier integrity, and host–microbiota interactions, while highlighting recent advances and existing gaps in the literature.

This narrative review summarizes the current evidence on dietary interventions, ultra-processed foods, and food additives in Crohn’s disease. A non-systematic literature search was performed using PubMed, Scopus, and Web of Science to identify relevant articles published up to January 2025. Search terms included combinations of Crohn’s disease, diet, nutrition, ultra-processed foods, food additives, emulsifiers, artificial sweeteners, food colorants, and nanoparticles. Additional relevant studies were identified through manual screening of reference lists. Articles were selected based on their relevance to the scope of this review.

## 2. Nutritional Strategies for CD Management

In addition to the role of diet in the pathogenesis of CD, several nutritional strategies have been shown to improve intestinal inflammation and disease-related symptoms (Table 1).

### 2.1. Crohn’s Disease Exclusion Diet (CDED)

The Crohn’s disease exclusion diet (CDED) is a structured, evidence-based dietary therapy developed to induce and maintain remission in patients with CD. It is particularly designed to reduce exposure to certain dietary components that may trigger inflammation and intestinal dysbiosis. CDED combines specific whole foods with partial enteral nutrition (PEN) to provide the necessary caloric intake and essential nutrients [16].

The CDED regimen is divided into an induction phase and a maintenance phase. In the induction phase, which covers the first 6 weeks, patients consume a strict combination of whole foods with 50% PEN to ensure adequate caloric intake. In the maintenance phase, which covers the following 6 weeks, PEN is reduced to 25%, and the diet is gradually expanded with additional food options while continuing to exclude problematic dietary triggers, such as processed meats, emulsifiers, artificial sweeteners, and foods high in saturated fats and simple sugars [17].

Multiple studies have assessed the efficacy of the CDED in pediatric patients with CD.

In a randomized controlled trial (RCT) involving 78 pediatric patients, CDED plus 50% PEN was better tolerated than exclusive enteral nutrition (EEN) and achieved 75% clinical remission versus 59% in the EEN group at week 6. At week 12, the CDED group outperformed the EEN group in all clinical parameters [18]. A post hoc analysis of the Levine 2019 RCT showed that both groups (CDED and EEN) achieved rapid responses at week 3 (82% and 85%, respectively) [18,19].

In a retrospective study involving 15 pediatric patients, 100% remission was achieved at weeks 6 and 12 with CDED plus PEN, with 87% of treatment-naïve patients maintaining remission at week 24 [20]. In another randomized controlled trial, a short course of EEN followed by CDED combined with PEN was compared with prolonged EEN. Although the study was underpowered to demonstrate superiority, the combined approach was effective in maintaining remission, suggesting that an early transition to CDED may represent a feasible and patient-friendly strategy [21].

While initial research focused on pediatric populations, recent studies have investigated the efficacy of CDED in adult patients.

A Polish study involving 32 adult patients with mild-to-severe CD demonstrated that 76.7% achieved clinical remission after 6 weeks of CDED plus PEN, which increased to 82.1% after 12 weeks. FCP levels significantly improved from baseline [22]. In an open-label, randomized trial of 44 adult patients with mild-to-moderate CD, CDED plus PEN resulted in 68% remission at week 6, while CDED alone achieved 57% remission. At week 24, 80% of those in remission maintained their remission status [17]. In a pilot study examining adults with pouchitis, 66.7% achieved remission at week 6, 60% at week 12, and 46.7% maintained remission at week 24 following the CDED plus PEN intervention [17]. More recently, a randomized controlled trial in 60 pediatric patients with mild-to-moderate Crohn’s disease directly compared CDED alone to CDED plus 50% partial enteral nutrition. After 8 weeks, both strategies achieved comparable clinical response and remission rates (>60%), with similar improvements in disease activity, inflammatory markers, fecal calprotectin, and nutritional status. Although the addition of partial enteral nutrition was associated with a better quality of life, it did not improve clinical efficacy, suggesting that CDED alone may represent an effective induction strategy in selected pediatric patients [23].

Recent evidence further supports the role of CDED as an established nutritional therapy for mild-to-moderate CD. A 2026 expert review highlighted its applicability across different age groups, disease phenotypes, and healthcare settings, emphasizing the importance of personalization, cultural adaptation, adherence strategies, and multidisciplinary management [24].

In addition, a 2026 scoping review summarized the expanding clinical use of CDED and confirmed its relevance as part of an integrated therapeutic approach, while also underlining the need for further studies on long-term outcomes, severe disease phenotypes, and combination strategies with biologic therapy [25]. Recent pediatric data suggest that Mediterranean-adapted versions of CDED may represent a promising and more sustainable approach, particularly in children with mild Crohn’s disease [26]. Although CDED currently represents the dietary intervention supported by the strongest evidence, several other nutritional approaches have also been investigated, each targeting different aspects of Crohn’s disease pathophysiology.

### 2.2. Specific Carbohydrate Diet (SCD)

Another commonly used therapeutic diet for CD is the specific carbohydrate diet (SCD). The SCD was popularized by Elaine Gottschall in her book *Breaking the Vicious Cycle* [27]. This dietary regimen emphasizes the exclusion of complex carbohydrates and focuses on easily digestible, simple carbohydrates. By restricting certain food groups, the SCD aims to reduce bacterial overgrowth, improve gut health, and minimize inflammation.

The SCD allows fresh fruits and vegetables, with the exception of starchy options like potatoes and yams. Some legumes (e.g., lentils and split peas) are permitted, while others (e.g., chickpeas and soybeans) are excluded. Grains are strictly prohibited. Sweeteners like saccharin and honey are acceptable, but processed sugars and artificial sweeteners are avoided. Canned fruits and vegetables are excluded due to potential added sugars and starches. While unprocessed meats are allowed, processed, canned, and smoked meats are restricted. Milk is excluded, but cheeses with minimal lactose and homemade yogurt fermented for 24 h are permitted [27].

Most studies examining the efficacy of the SCD have been conducted in pediatric populations. For example, Suskind et al. have provided promising evidence that the SCD or modified versions can improve CD symptoms and reduce inflammation. For instance, a small, randomized trial comparing the SCD, a modified SCD, and a whole-food (WF) diet showed symptom improvement and reduced systemic inflammation markers in the SCD and modified SCD groups. However, fecal calprotectin levels, an intestinal marker of inflammation, did not improve significantly in the SCD group by week 12 [28].

In a study by Cohen et al., 10 children with CD who followed the SCD as primary therapy were evaluated. Four patients achieved complete endoscopic healing, as assessed via video capsule endoscopy, while six patients experienced clinical remission [29].

In a randomized controlled trial investigating dietary interventions for pediatric CD, patients following the SCD, a modified SCD, and a whole-food diet achieved clinical remission by week 12. Notably, CRP levels improved across all dietary groups, with the greatest reduction seen in the modified SCD group [30].

In contrast to pediatric studies, research on the SCD in adults is more limited.

In a recent randomized trial comparing the SCD to the MD in adults with CD, no significant difference was observed in achieving symptomatic remission at week 6 (SCD 46.5% vs. MD 43.5%; *p* = 0.77). Both diets had comparable effects on fecal calprotectin and CRP responses. Given the greater ease of following the MD and its additional health benefits, the study suggests that the MD may be preferable for most patients with mild-to-moderate CD symptoms [31].

Despite its potential benefits, the SCD is a complex dietary regimen that requires rigorous planning, careful ingredient selection, and dedication to food preparation. Patients must adhere strictly to the dietary restrictions for at least one year to experience substantial improvements in symptoms and inflammatory markers such as the erythrocyte sedimentation rate (ESR), fecal calprotectin, and CRP.

Unlike the highly restrictive SCD, other dietary strategies focus primarily on symptom control rather than disease modification.

### 2.3. Low-FODMAP Diet

The low-FODMAP diet is another nutritional strategy that has gained attention for its role in improving gastrointestinal symptoms in patients with Crohn’s disease (CD). This diet restricts foods rich in fermentable oligosaccharides, disaccharides, monosaccharides, and polyols (FODMAPs), which are poorly absorbed carbohydrates that ferment in the gut, leading to gas production, bloating, and abdominal discomfort [32].

More recent evidence has clarified that the low-FODMAP diet should not be considered a primary anti-inflammatory treatment for CD but rather a targeted approach for the management of functional gastrointestinal symptoms, particularly in patients in remission or with overlapping irritable bowel syndrome-like symptoms [33].

Several recent systematic reviews and meta-analyses confirm that this dietary strategy is effective in reducing abdominal pain, bloating, and diarrhea, while its effects on objective inflammatory markers remain limited and inconsistent [34,35].

In a randomized controlled trial involving patients with quiescent CD and persistent gastrointestinal symptoms, 52% of patients following the low-FODMAP diet reported adequate symptom relief, compared to 16% in the control group, although no significant improvements were observed in inflammatory markers such as fecal calprotectin or C-reactive protein [36].

Emerging data have also raised concerns regarding its impact on the gut microbiota. The restriction of fermentable carbohydrates may reduce beneficial bacterial populations, particularly Bifidobacteria, and decrease the production of short-chain fatty acids, which are essential for intestinal health [36].

For this reason, current recommendations emphasize that the low-FODMAP diet should be implemented as a short-term intervention, followed by a structured reintroduction phase under the supervision of a trained nutritionist to minimize the risk of dysbiosis and nutritional deficiencies.

Overall, while the low-FODMAP diet represents an effective strategy for symptom control and improvement in quality of life in selected patients with CD, it should not be considered a first-line or disease-modifying treatment, and its long-term use requires careful monitoring.

In contrast to restrictive diets developed for symptom management, broader dietary patterns such as the Mediterranean diet aim to promote long-term intestinal health through modulation of inflammation and the gut microbiota.

### 2.4. Mediterranean Diet (MD)

The Mediterranean diet (MD) has emerged as one of the most promising and sustainable dietary strategies for the management of Crohn’s disease (CD). Characterized by a high intake of fruits, vegetables, whole grains, legumes, fish, and extra virgin olive oil, with limited consumption of red meat and processed foods, the MD is associated with anti-inflammatory effects and the promotion of a healthy gut microbiota [37].

A randomized controlled trial comparing the specific carbohydrate diet (SCD) to the Mediterranean diet (MD) in adults with mild-to-moderate CD showed comparable efficacy in achieving symptomatic remission [31]. Another study conducted by Chicco et al. explored the impact of the MD on nutritional status, liver steatosis, and clinical outcomes in IBD patients. After following the MD for six months, patients with CD showed improvements in both BMI and waist circumference, along with a significant reduction in the prevalence of liver steatosis. Moreover, patients adhering to the MD experienced fewer active disease episodes and lower levels of inflammatory biomarkers such as CRP and fecal calprotectin [38].

In a single-center observational study, Cadoni et al. evaluated MD adherence in patients with IBD. The study highlighted that while IBD patients tend to have lower diet quality scores compared to healthy individuals, those with higher adherence to the MD showed better inflammatory profiles and improved quality of life. Interestingly, patients with ileocolonic stricturing CD had lower MD adherence scores, suggesting that acceptance of the MD may vary across different disease subtypes [39].

A recent editorial highlighted the potential of a modified version of the Mediterranean diet, termed IBDMED, specifically adapted to the nutritional requirements of patients with Crohn’s disease. This dietary pattern emphasizes the consumption of fermented foods, a wide variety of plant-based products, and colorful fruits and vegetables to promote microbial diversity and enhance short-chain fatty acid production. Evidence indicates that patients adhering to the IBDMED demonstrated improved inflammatory profiles, lower CD Activity Index (CDAI) scores, and greater gut microbiota diversity [40]. In line with these findings, more recent evidence has further strengthened the role of the Mediterranean diet in CD.

Overall, the MD emerges as a beneficial and sustainable dietary strategy for patients with CD. It not only controls symptoms and sustains remission but also improves nutritional status, reduces inflammation, and enhances gut microbiota health. Due to its flexibility, anti-inflammatory properties, and alignment with general healthy eating principles, the MD should be considered a primary dietary approach for CD management, personalized to meet individual needs and disease stages.

### 2.5. Anti-Inflammatory Diet for IBD (IBD-AID)

The anti-inflammatory diet for IBD (IBD-AID) reduces intestinal inflammation in IBD by avoiding highly processed or refined carbohydrates and lactose, which are responsible for the growth of pro-inflammatory pathogenic microbes. This diet is supported by the introduction of prebiotics and probiotics, such as fresh yogurt, fermented vegetables, and kefir. However, the conclusions of Lichtenstein’s review differ, as it reported that treatment with prebiotics and probiotics used in the IBD-AID—with the exception of *Saccharomyces boulardii*—did not demonstrate efficacy in promoting or maintaining clinical remission in patients with CD [41]. Another important point is the reduction in total and saturated fats, with a preference for vegetable oils and omega-3s, while still limiting dairy products such as aged cheeses and yogurt. The diet starts with a soft consistency and gradually moves to a more solid one [42].

In the prospective EPIC-IBD cohort study by Andersen et al., no associations were observed between the intake of grains, vegetables, fruit-derived fiber, or total fiber and the risk of developing CD [43].

In a study by Morton et al., foods commonly avoided by patients with Crohn’s disease were evaluated. The authors were unable to identify a specific group of foods consistently associated with disease exacerbation. Instead, the findings suggest that food avoidance is largely driven by the occurrence of gastrointestinal symptoms, such as bloating and abdominal pain, which vary considerably among individuals. Therefore, these dietary restrictions appear to reflect patient-specific symptom management rather than objective evidence that these foods directly promote intestinal inflammation or disease activity [44].

**Table 1:** Dietary approaches in Crohn’s disease.

Diet	Key Components	Mechanism of Action	Clinical Evidence	Limitations
CDED (Crohn’s Disease Exclusion Diet)	Whole foods, exclusion of additives and processed foods, partial enteral nutrition	Reduces exposure to dietary triggers and food additives; improves microbiota composition and barrier function	Strong evidence in pediatric and adult CD; higher remission and adherence compared to EEN [17,18,19,20,21,22]	Requires structured protocol and dietitian supervision
EEN (Exclusive Enteral Nutrition)	Liquid formula-based diet	Reduces antigen exposure and modulates gut microbiota	Effective for inducing remission, especially in children [18,21]	Poor long-term adherence; limited palatability
SCD (Specific Carbohydrate Diet)	Excludes complex carbohydrates, grains, processed foods	Reduces bacterial fermentation and dysbiosis	Improves symptoms; mixed results on inflammatory markers [27,28,29,30]	Highly restrictive; limited adult evidence
Low-FODMAP Diet	Restriction of fermentable carbohydrates	Reduces gas production and luminal distension	Effective for symptom relief (bloating, pain); limited effect on inflammation [33,34,35]	Not suitable for long-term use; may reduce beneficial microbiota
Mediterranean Diet (MD)	High in fiber, fruits, vegetables, olive oil, and fish	Anti-inflammatory effects; promotes SCFA production and microbiota diversity	Comparable to SCD for symptom control; beneficial metabolic effects [30,37,38,39]	Requires adherence; evidence in CD still emerging
IBDMED	Modified MD with emphasis on fermented foods and plant diversity	Enhances microbial resilience and anti-inflammatory pathways	Early evidence suggests improvement in disease activity and microbiota diversity [39]	Limited clinical data; not yet standardized

## 3. Impact of Ultra-Processed Foods (UPFs) on Crohn’s Disease

An increased incidence of CD is also associated with the consumption of ultra-processed foods (UPFs), which are high in energy density and abundant in sugars, salt, and unhealthy fats, while being deficient in dietary fiber, vitamins, minerals and proteins. UPFs are harmful to our bodies because they are obtained through industrial processes that alter their composition, contain unhealthy ingredients, and include additives to make them more palatable [45]. The intake of UPFs (industrial products such as ice cream, candy, spreads, margarine, energy bars, fast food, pastries, cakes, chocolate, cookies and soft drinks) is associated with an increased risk of IBD [46]. In a large prospective cohort study including 245,112 participants from 21 countries, Narula et al. (2021) reported that individuals with the highest UPF intake had a significantly higher risk of developing IBD compared with those consuming minimal amounts (hazard ratio [HR] = 1.82, 95% CI = 1.22–2.72) [47]. Similarly, a recent meta-analysis confirmed a dose–response relationship, showing that every 10% increase in daily energy intake from UPFs was associated with a 19% higher risk of CD [10]. Moreover, a U.S. cohort analysis found that participants in the highest quartile of UPF consumption had an approximately 70% higher risk of CD compared with the lowest quartile [48].

Mechanistically, UPFs may directly impair intestinal function by reducing mucus production and altering goblet cell activity. Indirectly, they can promote dysbiosis, leading to thinning of the mucus layer and increased intestinal permeability due to bacterial translocation and inflammation. Furthermore, the presence of advanced glycation end products (AGEs), formed during high-temperature processing, can further compromise mucus integrity, intestinal permeability, and inflammatory balance [49,50], thereby favoring an allergic response to food antigens [51]. However, when assessing the impact of UPFs on the development of CD, it is crucial to consider potential age-related differences, cumulative exposure effects, and epigenetic interactions that may vary by ethnicity. Patients with CD have been shown to consume significantly greater—sometimes even double—amounts of UPFs compared to healthy controls, although it remains unclear whether this dietary pattern precedes disease onset or reflects post-diagnostic changes in eating behavior related to gastrointestinal symptoms [52].

More recent evidence has further strengthened the association between UPF intake and CD outcomes. In a prospective cohort study of 111 adults with CD in steroid-free clinical remission, high UPF consumption was associated with a significantly increased risk of clinical relapse over 12 months (HR = 3.86; 95% CI: 1.30–11.47), with ultra-processed breads, pastries, starches, oils, and spreads showing the strongest associations [53]. Importantly, relapse was accompanied by increased fecal calprotectin levels, suggesting that UPF intake may be linked not only to symptom recurrence but also to objective inflammatory activity. Recent evidence from large-scale prospective analyses and meta-analyses further supports the association between UPF-rich dietary patterns and increased CD risk, while minimally processed and Mediterranean-like dietary patterns appear protective [47,54].

A 2026 systematic review and meta-analysis including more than 1.5 million participants confirmed that high consumption of ultra-processed foods is associated with an increased risk of inflammatory bowel disease overall and, more specifically, with Crohn’s disease, whereas no significant association was observed for ulcerative colitis. Furthermore, a recent patient-based, multi-omics study demonstrated that habitual UPF intake is associated with gut dysbiosis, pro-inflammatory metabolomic signatures, and unfavorable clinical characteristics, providing biological evidence supporting the role of UPFs in Crohn’s disease pathogenesis [55].

## 4. Impact of Food Additives on Crohn’s Disease

Food additives are substances with no nutritional value added to food to improve its physical appearance and shelf life. Although they are considered non-mutagenic and non-toxic compounds, their potential long-term impact on the intestinal immune system is currently unclear. Recently, neurotoxic, cardiovascular, tumorigenic [56] and allergenic effects [57] have been suggested, especially with regard to synthetic additives.

**Figure 1:**
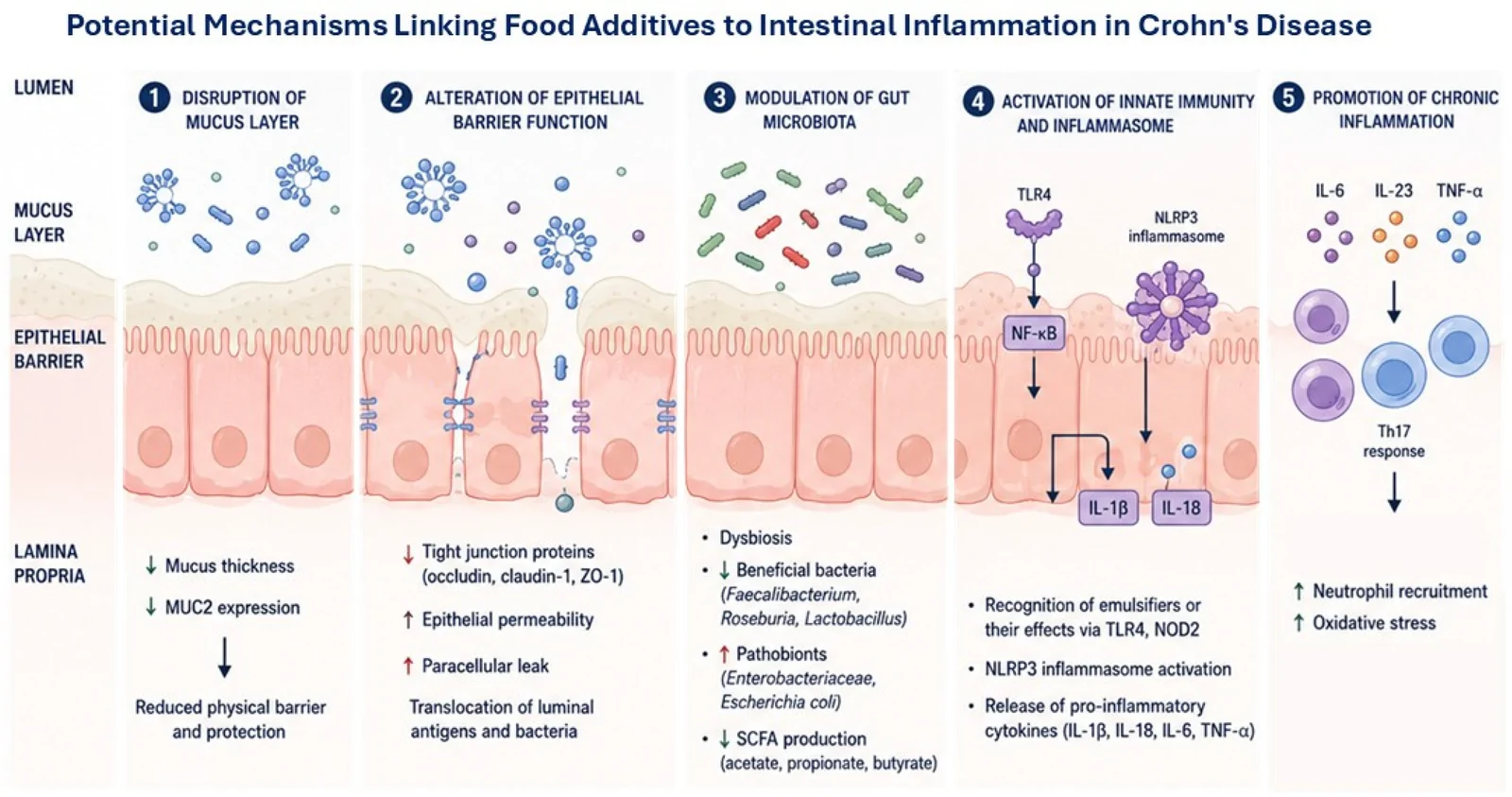
Proposed mechanisms by which food additives may contribute to intestinal inflammation in Crohn’s disease. Food additives may impair intestinal homeostasis through multiple interconnected mechanisms, including disruption of the mucus layer, increased epithelial permeability due to tight junction dysfunction, gut microbiota dysbiosis, activation of innate immune pathways, and enhanced production of pro-inflammatory cytokines, ultimately promoting chronic intestinal inflammation. Green arrows indicate decreased expression or abundance, whereas red arrows indicate increased expression or activity. Abbreviations: MUC2, mucin 2; ZO-1, zonula occludens-1; TLR4, Toll-like receptor 4; NF-κB, nuclear factor kappa B; NLRP3, NOD-like receptor family pyrin domain containing 3; IL, interleukin; TNF-α, tumor necrosis factor-α; Th17, T helper 17.

Most available evidence on food additives derives from in vitro and animal studies, which suggest that these compounds may disrupt gut homeostasis through multiple mechanisms, including alterations in gut microbiota composition, reduced microbial diversity, and impairment of intestinal barrier function (Table 2, Figure 1). These changes may contribute to dysbiosis, a key feature of CD. However, human data remain limited, and the clinical relevance of these findings is still under investigation [58].

An important aspect to consider is cumulative exposure. Food additives are widely present in ultra-processed foods, and their frequent consumption may lead to intake levels that approach or exceed recommended thresholds, particularly in individuals adhering to Western dietary patterns [59].

Overall, while food additives represent a plausible environmental factor in the pathogenesis and progression of Crohn’s disease, their effects are likely influenced by dosage, dietary context, and host-specific factors, including baseline microbiota composition and disease status.

IBD patients are more sensitive than healthy individuals to the effects of additives; in fact, polysorbate 80 (P80), sucralose (SUC), maltodextrin (MDX), sodium nitrite (Nit) and titanium dioxide (TiO_2_) exert a greater negative effect on bacteria such as *F. prauznitzii* (butyrate producer) and *Roseburia* (anti-inflammatory regulatory T-cell producer), which are typically reduced in IBD. In addition, the first three influence the microbiota of healthy patients by making it similar to that of IBD patients [60].

### 4.1. Emulsifiers

Emulsifiers are compounds capable of making substances miscible by the presence of a lipophilic and a hydrophilic end; therefore, they are added to foods to improve their consistency, shelf life, and palatability. They are commonly labeled E400 to E499 [61]. Beyond their technological use, emulsifiers have been implicated in compromising intestinal barrier integrity, promoting dysbiosis and enhancing low-grade chronic inflammation [59,62].

Among synthetic emulsifiers, carrageenan (E407), carboxymethyl cellulose (CMC, E466) and P80 (E433) are the most extensively studied. Both compounds have shown the ability to alter gut microbiota composition irreversibly, reduce microbial diversity, and promote mucosal inflammation [63]. Their action includes disruption of the mucosal layer, increased epithelial apoptosis, and enhanced permeability, thereby mimicking core pathological features of CD [62]. In vitro studies show that P80 and CMC reduce the number of intact microbial cells in the gut microbiome, with effects comparable to antibiotics, likely due to their surfactant action that disrupts bacterial membranes [64]. Moreover, Loayzaa et al. found that these emulsifiers promote the growth of *Proteus* spp. and Negativicutes, microbial taxa associated with CD recurrence [65]. Recent human evidence has begun to confirm these findings. In a randomized, placebo-controlled trial, supplementation with dietary emulsifiers was associated with reduced short-chain fatty acid production and increased intestinal permeability, although no significant changes in systemic inflammatory markers were observed over the short term [66].

Importantly, emerging clinical data suggest that reducing emulsifier intake may have therapeutic benefits. In the multicenter ADDapt trial, patients with CD following a low-emulsifier diet showed higher rates of clinical response and remission, along with significant reductions in fecal calprotectin, compared with controls [67].

Further support for the clinical relevance of food additives comes from recent human studies in which emulsifiers and artificial sweeteners were directly quantified in biological samples from patients with Crohn’s disease. In this context, higher levels of polysorbate-80-derived metabolites were associated with increased intestinal permeability and greater disease activity, suggesting a potential synergistic interaction between different classes of food additives [68].

Carrageenan (E407), a sulfated polysaccharide derived from red algae, is widely used as a thickening agent in processed foods. Preclinical studies suggest that carrageenan may promote dysbiosis, increase pro-inflammatory cytokine production, and impair mucosal integrity. Its degradation into low-molecular-weight sulfated compounds within the gastrointestinal tract may further contribute to inflammatory processes through interactions with sulfate-reducing bacteria [63,69,70]. However, it should be noted that most of these findings derive from animal models, and human evidence remains limited.

Other emulsifiers, including agar-agar, diacetyl tartaric acid esters of mono- and diglycerides (DATEM), and hydroxypropyl methylcellulose, have been less extensively studied but may similarly influence gut microbiota composition and diversity [63].

In contrast, not all emulsifiers exert detrimental effects. Some naturally occurring compounds, such as soy and rapeseed lecithin, have shown neutral or potentially beneficial effects in certain contexts, including the promotion of butyrate-producing bacteria. These findings suggest that the biological impact of emulsifiers is highly heterogeneous and influenced by factors such as chemical structure, dosage, dietary context, and host microbiota composition [65,71].

### 4.2. Preservatives

Preservatives are additives used to extend the shelf life of food products by inhibiting the growth of microorganisms and are classified within the E200–E299 category. Beyond their antimicrobial function, increasing evidence suggests that certain preservatives may influence gut homeostasis through both direct and microbiota-mediated mechanisms [61]. Compounds such as nitrites, sulfites, sorbates, and benzoates have been shown to exert non-selective antimicrobial effects, potentially reducing beneficial commensal bacteria and leading to decreased microbial diversity and altered short-chain fatty acid production, both of which are essential for maintaining intestinal homeostasis. In addition, these compounds may induce oxidative stress by increasing reactive oxygen species (ROS), thereby impairing epithelial barrier integrity and promoting mucosal inflammation [58]. Recent evidence has further supported these observations. In an in vitro fecal fermentation study including adults with CD and healthy controls, several food additives—including preservatives—were shown to significantly alter gut microbial composition and fiber fermentation capacity, suggesting that patients with Crohn’s disease may exhibit increased susceptibility to additive-induced microbiome perturbations [72].

Despite these mechanistic insights, most available data remain preclinical or derived from ex vivo models, and the clinical relevance of preservatives in the pathogenesis or progression of Crohn’s disease is still not fully established [73,74].

### 4.3. Colorants

Food colorants are additives used to enhance or restore the color of food products and are classified within the E100–E199 category. They may be of natural or synthetic origin. Although human evidence remains limited, recent mechanistic studies have highlighted microbiota-dependent pathways through which certain synthetic dyes may contribute to intestinal inflammation [61]. In particular, Red 40 and Yellow 6 have been shown to be metabolized by commensal bacteria, including *Bacteroides ovatus* and *Enterococcus faecalis*, into bioactive compounds such as 1-amino-naphthol-6-sulfonate. This metabolite induces the expression of pro-inflammatory cytokines, including interleukin-23 (IL-23), thereby activating Th17-mediated immune pathways implicated in Crohn’s disease pathogenesis [75]. Sudan colorants and their metabolites are deleterious to the normal growth of the intestinal microbiota, as are nanoparticles of TiO_2_ (white colorant), which, in addition to altering the composition of the intestinal flora, also change its function and destroy the integrity of the mucosa. Although azoic Sudan colorants have been banned, unfortunately, they are still illegally used due to their low cost [58].

Recent evidence has further expanded this concept, suggesting that the biological effects of food colorants may be mediated by microbiota-dependent toxicity, in which bacterial metabolism generates pro-inflammatory intermediates that influence host immune responses [75].

In addition, increasing attention has been given to colorant-related nanoparticles, particularly TiO_2_ (E171). Experimental studies have shown that TiO_2_ nanoparticles can disrupt gut microbiota composition, impair mucosal barrier integrity, and activate innate immune pathways, including the NLRP3 inflammasome, leading to increased production of interleukin-1β (IL-1β) and enhanced inflammatory signaling [76].

Further studies suggest that TiO_2_ may undergo physicochemical transformations within the gastrointestinal tract, modifying its interaction with microbial communities and host tissues and contributing to dysbiosis and immune dysregulation [77].

Overall, while these findings support a potential role for food colorants in modulating intestinal inflammation, current evidence remains largely preclinical, and well-designed human studies are required to determine their clinical relevance in Crohn’s disease.

### 4.4. Antioxidants

Antioxidants (E300–E399) are used in the food industry to reduce the oxidation of fats in food products. They can be beneficial to human health, although synthetic ones may be harmful [61]. Natural antioxidants, such as phenolic acids and flavonoids, are present in fruits, vegetables, spices and honey. In mouse models, they reduce inflammation by reducing cytokines and markers of oxidative stress and apoptosis. Beyond their effects on oxidative stress, they can act as prebiotics [50]. In some studies, an increase in Lactobacillus and Akkermansia and a reduction in pro-inflammatory bacteria involved in IBD were observed [71].

Some clinical evidence exists, although it remains limited. A study evaluating curcumin supplementation in patients with inflammatory bowel disease demonstrated improvements in oxidative stress markers, cytokine levels, and fecal calprotectin, supporting its potential role as an adjunctive therapy [78].

More recent evidence further supports the potential role of antioxidants in IBD. A systematic review of randomized controlled trials concluded that curcumin may improve clinical outcomes when used as adjunctive therapy, although results remain heterogeneous and not specific to CD alone [79].

Overall, antioxidants appear to exert protective effects through both the direct reduction of oxidative stress and the indirect modulation of the gut microbiota. Nevertheless, their clinical role in CD remains supportive rather than therapeutic, and further well-designed human studies are needed to clarify their efficacy.

### 4.5. Sweeteners

Sweeteners are widely used in the food industry to impart a sweet taste without necessarily increasing the caloric load. They include both natural compounds (e.g., stevia and sugar alcohols) and synthetic agents (e.g., aspartame, saccharin, and sucralose). Although generally considered safe for the general population, increasing evidence suggests that their interaction with the gut microbiota and immune system may have important implications in CD [61].

Epidemiological data indicate that the regular consumption of sugar-sweetened beverages is associated with an increased risk of CD, whereas similar associations have not been consistently observed for artificially sweetened beverages. However, these findings may be influenced by study design and confounding factors and do not necessarily reflect the biological effects of individual sweeteners [80]. The impact of sweeteners on gut homeostasis appears to be highly heterogeneous and compound-specific. Some agents, such as polyols, may exert prebiotic-like effects by promoting microbial diversity and reducing inflammation. In contrast, artificial sweeteners such as sucralose and saccharin have been associated with increased intestinal permeability, altered microbial composition, and enhanced inflammatory responses in experimental models. These effects may be mediated by microbiota-dependent mechanisms, leading to the production of pro-inflammatory metabolites and activation of immune pathways [58].

Recent studies have further demonstrated that artificial sweeteners may interfere with bacterial metabolic activity and quorum sensing, suggesting that their effects extend beyond compositional changes to functional alterations of the gut microbiome [81]. In addition, certain sweeteners, including sucralose and aspartame, have been shown to increase pro-inflammatory cytokines such as IL-6, IL-1β, and TNF-α in a dose-dependent manner, further supporting their potential role in intestinal inflammation [82].

Recent human evidence has significantly strengthened these observations. In the ENIGMA study, artificial sweeteners were directly quantified in biological samples from patients with Crohn’s disease and healthy controls, and higher levels were associated with increased disease activity, including the Crohn’s Disease Activity Index and fecal calprotectin. Notably, interactions between artificial sweeteners and emulsifier-derived metabolites were identified, suggesting a synergistic effect on intestinal permeability and inflammation [68].

Additional evidence suggests that the sugar alcohol sorbitol may exacerbate disease-related complications. For example, sorbitol has been shown to promote *Clostridioides difficile* infection in vivo, which may worsen disease severity in IBD patients [83]. Furthermore, artificial sweeteners such as steviol may stimulate the release of pro-inflammatory cytokines and increase exposure to bacterial components such as lipopolysaccharide (LPS), further contributing to mucosal inflammation [71].

Basson et al. found that IBD patients tend to prefer sweet flavors and artificially sweetened products, assuming them to be healthier. However, accumulating evidence indicates that many artificial sweeteners can disrupt gut microbial composition and function, promoting dysbiosis and inflammation [84]. In light of this, even the World Health Organization (WHO) no longer recommends the use of non-sugar sweeteners for weight control, due to emerging data on metabolic and inflammatory consequences [85]. Like emulsifiers, the biological effects of sweeteners are based on dosage and frequency of consumption, influenced by the existing microbiota composition and mucosal health of the host, and may differ between healthy individuals and those with IBD. While some compounds show promise in modulating the gut positively (e.g., polyols), others may be harmful in the context of chronic inflammation and mucosal damage.

### 4.6. Flavor Enhancers

Flavor enhancers (E600–E699) are used to improve the palatability of food products. Although their role in CD has been less extensively studied compared with other additives, emerging evidence suggests that certain compounds may influence intestinal inflammation and immune responses [61]. High dietary sodium chloride intake has been shown to promote intestinal inflammation in experimental models. This effect is mediated not only by alterations in the gut microbiota—such as reduced *Lactobacillus* abundance and decreased short-chain fatty acid production—but also by direct modulation of the immune system. In particular, high salt intake has been associated with increased differentiation of pro-inflammatory Th17 cells and reduced regulatory T-cell activity, leading to enhanced production of cytokines such as IL-17 and IL-23, which are central to CD pathogenesis [86,87]. Lactobacilli communities are particularly influenced by NaCl, with consequences for gut immunity [88]. Monosodium glutamate (MSG), another commonly used flavor enhancer, has also been investigated for its potential effects on gut physiology. Experimental studies suggest that MSG may influence intestinal metabolism and barrier function, although current evidence remains limited, and further research is needed to clarify its role in CD [73].

### 4.7. Nanoparticles

Attention should also be given to nanoparticles (NPs) with sizes ranging from 1 to 100 nm. These substances are used as food additives, such as silver (E 174) and TiO_2_ (E 171) for colorants, especially in the confectionery industry, and are incorporated into various food packaging materials due to their barrier capacity against oxygen, moisture and carbon dioxide, as well as for their antimicrobial activity. These NPs can be released into food and, therefore, once ingested, have effects, especially in the intestines. Although their effects in humans have not been extensively studied, studies to date indicate their ability to induce dysbiosis [89].

Emerging evidence suggests that nanoparticles may affect gut homeostasis through multiple mechanisms. Experimental studies have shown that ingested nanoparticles can alter gut microbiota composition, reduce microbial diversity, and impair mucosal barrier integrity. In addition, nanoparticles may activate innate immune pathways, including the NLRP3 inflammasome, leading to increased production of pro-inflammatory cytokines such as IL-1β and enhanced inflammatory signaling [90].

Recent studies have also highlighted nanoparticle–microbiota interactions, wherein nanoparticles can bind to bacterial surfaces and alter microbial metabolism and function, contributing to dysbiosis and immune dysregulation. However, despite growing mechanistic evidence, human data remain limited, and the clinical relevance of nanoparticle exposure in CD is still not fully established [91].

**Table 2:** Impact of food additives on Crohn’s disease.

Additive Category	Common Examples	Use in the Food Industry	Effects on Intestinal Health and CD
Emulsifiers	Polysorbate 80 (P80), carboxymethylcellulose (CMC), sunflower lecithin, carrageenan, agar-agar, HPMC, DATEM, glyceryl oleate, soy lecithin, glycerol monolaurate	Improve texture, stability, and shelf life (e.g., sauces, ice cream, dressings, plant-based milks)	Promote dysbiosis, increase intestinal permeability, disrupt mucosal layer, alter goblet cell function, increase inflammation; damage comparable to antibiotics; effects depend on type, dose, and host condition [60,61,62,63,64]
Preservatives	Nitrites, nitrates, sodium benzoate, sorbates, sulfites, silver nanoparticles	Extend shelf life, antimicrobial function in processed meats, canned food, baked goods	Alter gut microbiota composition, reduce microbial diversity, increase oxidative stress and inflammation; sulfites may exceed daily safe limits in Western diets; Ag NPs interfere with beneficial bacteria and immune function [56,70,71,72]
Food Colorants	Tartrazine (E102), Allura Red (E129), Brilliant Blue (E133), titanium dioxide (E171), Sudan dyes	Enhance the color and visual appeal of drinks, candies, processed snacks	May increase intestinal inflammation and IL-23 levels; some require microbiota metabolism to become harmful; TiO_2_ and Sudan dyes disrupt microbiota and damage the mucosa; illegal Sudan dyes still used due to low cost [73,74,75]
Artificial Sweeteners	Aspartame, sucralose, saccharin, acesulfame K, sorbitol, steviol, sucrose, polyols (mannitol, xylitol, isomalt, erythritol)	Sugar substitutes in diet products, soft drinks, sugar-free gums	Heterogeneous effects: some beneficial (polyols), others promote dysbiosis, permeability, cytokine release; ENIGMA: associated with disease activity; microbiota-mediated and synergistic effects with emulsifiers [79,80,81]
Antioxidants	Butylated hydroxyanisole (BHA), butylated hydroxytoluene (BHT), tocopherols (Vit E), polyphenols	Prevent oxidation of fats and oils in snacks, oils, baked goods	Synthetic antioxidants may disrupt gut metabolism; natural ones (polyphenols, flavonoids) show anti-inflammatory, antioxidant and prebiotic activity; promote Lactobacillus and Akkermansia, reduce pro-inflammatory bacteria [49,77,78]
Flavor Enhancers	Monosodium glutamate (MSG), sodium chloride (NaCl)	Enhance palatability in processed soups, snacks, seasonings	High salt promotes Th17 response, reduces Lactobacillus and SCFA; MSG may alter metabolism and barrier function; limited CD-specific evidence [85,86,87]
Nanoparticles	Silver (E174), titanium dioxide (E171)	Improve food texture, whiteness and packaging performance (colorants, barrier agents)	Induce dysbiosis, impair barrier, activate innate immunity (NLRP3 inflammasome); microbiota–particle interactions alter bacterial function [88,89,90]

## 5. Discussion

The findings of this review reinforce the central role of diet in modulating the course of CD, not only in symptom management but also in influencing the gut microbiota, mucosal integrity, and immune activation. While pharmacological therapies remain the mainstay of CD treatment, dietary interventions represent a non-invasive, modifiable strategy that can enhance patient outcomes and potentially reduce the disease burden over time.

Several dietary approaches have demonstrated clinical relevance. The CDED has shown promising results in both pediatric and adult populations, outperforming EEN in terms of adherence and long-term remission [17,20]. Similarly, the SCD and low-FODMAP diet have proven effective in reducing gastrointestinal symptoms and inflammatory markers [27,35], although the evidence remains stronger in children than in adults, and some inflammatory biomarkers (e.g., fecal calprotectin) do not always respond significantly [28,36]. The MD appears to offer a more sustainable, anti-inflammatory dietary pattern that supports gut microbiota diversity and overall nutritional balance [31,40,92]. Its high content of fiber, polyphenols, unsaturated fats, and antioxidants makes it an ideal baseline for most CD patients, especially during remission. The IBD-tailored version of the MD (IBDMED) further personalizes dietary choices to support microbial resilience and reduce inflammation [40].

A key theme emerging from this review is the negative impact of UPFs and their associated food additives. UPFs are not only calorically dense and nutritionally poor but also loaded with substances that interfere with gut integrity [46,52]. Additives such as emulsifiers, preservatives, colorants, artificial sweeteners, and nanoparticles have been shown—mainly in preclinical and animal studies—to induce dysbiosis, impair mucosal barriers, and promote inflammation, particularly in genetically predisposed or already sensitized individuals [52,60].

Among these, emulsifiers show the strongest and most consistent evidence. Preclinical data have long demonstrated their ability to increase intestinal permeability and alter microbial composition [61,62,71], and these findings are now increasingly supported by human studies. A recent randomized controlled trial demonstrated that dietary emulsifier exposure reduces short-chain fatty acid production and increases intestinal permeability [66]. Furthermore, the ADDapt trial showed that a low-emulsifier diet significantly improved clinical response and reduced fecal calprotectin in patients with CD [67].

Similarly, artificial sweeteners have gained attention as potential modulators of disease activity. While earlier studies were largely preclinical [51,60,63,81,82,83], recent human evidence has substantially strengthened this association. In the ENIGMA study, artificial sweeteners and emulsifier-derived metabolites were directly quantified in biological samples from patients with CD, and higher levels were associated with increased disease activity, including the CD Activity Index and fecal calprotectin [68]. Notably, this study also identified synergistic interactions between sweeteners and emulsifiers, suggesting that combined exposure may amplify intestinal permeability and inflammatory responses.

In contrast, the role of preservatives, colorants, and nanoparticles remains less clearly defined. Experimental studies indicate that these compounds may induce oxidative stress, alter microbiota composition, and activate innate immune pathways [58,59,61,75,89,93], including inflammasome signaling [90]. However, robust human data are still lacking, highlighting a critical gap in current research.

Another important aspect is patient behavior. Individuals with CD frequently adopt self-imposed dietary restrictions to manage symptoms, often avoiding fiber, dairy products, or fermentable carbohydrates [94]. While these strategies may provide short-term relief, they can lead to significant nutritional deficiencies, including vitamins (D, B12, and folate), minerals (iron, zinc, and calcium), and essential fatty acids [54,73]. Given the increased risk of malnutrition due to malabsorption and chronic inflammation, dietary interventions should be supervised by healthcare professionals to ensure nutritional adequacy [95].

From a practical perspective, the widespread consumption of UPFs in modern societies represents a major challenge. The complete elimination of these foods is often unrealistic [96,97]. Nevertheless, reducing UPF intake and prioritizing whole-food-based dietary patterns—such as the Mediterranean diet or CDED—may represent a feasible and effective strategy to improve disease outcomes and quality of life in CD patients.

Overall, current evidence supports a model in which diet influences CD through a complex interplay among nutrients, food additives, gut microbiota, and immune responses. Future research should focus on integrating dietary interventions into personalized treatment strategies, identifying biomarkers of dietary response, and clarifying the long-term clinical impact of reducing exposure to ultra-processed foods and food additives.

## 6. Conclusions

Diet represents a powerful, yet often underestimated, component in the management of Crohn’s disease. While pharmacological therapies remain the cornerstone of treatment, growing evidence indicates that dietary strategies can significantly influence disease activity, symptom burden, and intestinal homeostasis when integrated into a personalized and multidisciplinary approach.

The findings of this review highlight that both overall dietary patterns and specific food components play a critical role in modulating disease outcomes. Diets rich in fiber, polyphenols, and unsaturated fats support microbial diversity and anti-inflammatory pathways, whereas Western dietary patterns and ultra-processed foods are associated with dysbiosis, impaired barrier function, and increased inflammation.

Particular attention should be given to food additives, which are increasingly recognized as potential contributors to intestinal dysfunction. Emerging data suggest that certain additives, such as emulsifiers and artificial sweeteners, may disrupt gut barrier integrity, alter microbial composition, and promote inflammatory responses. These effects may be amplified by combined exposure to multiple additives, highlighting the importance of considering the overall dietary context rather than isolated compounds.

At the same time, dietary interventions must be carefully balanced to avoid unintended consequences. Patients with Crohn’s disease frequently adopt restrictive eating patterns that may alleviate symptoms but increase the risk of nutritional deficiencies. Therefore, dietary management should focus on individualized, evidence-based approaches that preserve nutritional adequacy while minimizing exposure to potentially harmful dietary factors.

From a practical perspective, the widespread consumption of ultra-processed foods in modern societies represents a significant challenge. While complete elimination may not be feasible, reducing their intake and prioritizing whole-food-based dietary patterns may offer a realistic and effective strategy to improve disease outcomes and quality of life.

Looking ahead, the integration of structured dietary counseling into routine clinical care should become a priority in Crohn’s disease management. Future research should aim to clarify the long-term impact of dietary interventions, better define the role of food additives in human disease, and explore the potential of personalized nutrition strategies as an adjunct to pharmacological therapy.

## Data Availability

No new data were created or analyzed in this study.
